# Antimicrobial Polyketide Metabolites from *Penicillium bissettii* and *P. glabrum*

**DOI:** 10.3390/molecules27010240

**Published:** 2021-12-31

**Authors:** Melissa M. Cadelis, Natasha S. L. Nipper, Alex Grey, Soeren Geese, Shara J. van de Pas, Bevan S. Weir, Brent R. Copp, Siouxsie Wiles

**Affiliations:** 1 School of Chemical Sciences, University of Auckland, Waipapa Taumata Rau, Private Bag 92019, Auckland 1142, New Zealand; natasha.nipper@gmail.com (N.S.L.N.); b.copp@auckland.ac.nz (B.R.C.); 2 Bioluminescent Superbugs Lab, School of Medical Sciences, University of Auckland, Waipapa Taumata Rau, Private Bag 92019, Auckland 1142, New Zealand; alex.grey@auckland.ac.nz (A.G.); s.geese@auckland.ac.nz (S.G.); s.vandepas@auckland.ac.nz (S.J.v.d.P.); 3 Manaaki Whenua, Landcare Research, Private Bag 92170, Auckland 1142, New Zealand; WeirB@landcareresearch.co.nz

**Keywords:** antimicrobial, penicillium, metabolite, fungi, natural products

## Abstract

Screening of several fungi from the New Zealand International Collection of Microorganisms from Plants identified two strains of *Penicillium, P. bissettii* and *P. glabrum*, which exhibited antimicrobial activity against *Escherichia coli,* *Klebsiella pneumoniae*, and *Staphylococcus aureus.* Further investigation into the natural products of the fungi, through extraction and fractionation, led to the isolation of five known polyketide metabolites, penicillic acid (**1**), citromycetin (**2**), penialdin A (**3**), penialdin F (**4**), and myxotrichin B (**5**). Semi-synthetic derivatization of **1** led to the discovery of a novel dihydro (**1a**) derivative that provided evidence for the existence of the much-speculated open-chained form of **1**. Upon investigation of the antimicrobial activities of the natural products and derivatives, both penicillic acid (**1**) and penialdin F (**4**) were found to inhibit the growth of Methicillin-resistant *S. aureus*. Penialdin F (**4**) was also found to have some inhibitory activity against *Mycobacterium abscessus* and *M. marinum* along with citromycetin (**2**).

## 1. Introduction

Manaaki Whenua’s International Collection of Microorganisms from Plants (ICMP) contains over 10,000 fungal cultures, most of which originate from Aotearoa New Zealand and the South Pacific [1]. These fungal cultures include a diverse range of fungal species, many of which are unique to Aotearoa New Zealand and most of which have not been rigorously tested for antimicrobial activity or investigated for novel natural products. The most well-known and significant example of antibiotic discovery from microbes was the discovery of penicillin from a *Penicillium rubens* culture by Alexander Fleming in 1928 [2,3]. The structure of Fleming’s penicillin was proposed in 1942 by Edward Abraham and confirmed by Dorothy Hodgkin in 1945. While the original penicillins were active mostly against Gram-positive bacteria, numerous semisynthetic antibiotics were produced in the subsequent years with an improved spectrum of activity [2].

**Figure 1:**
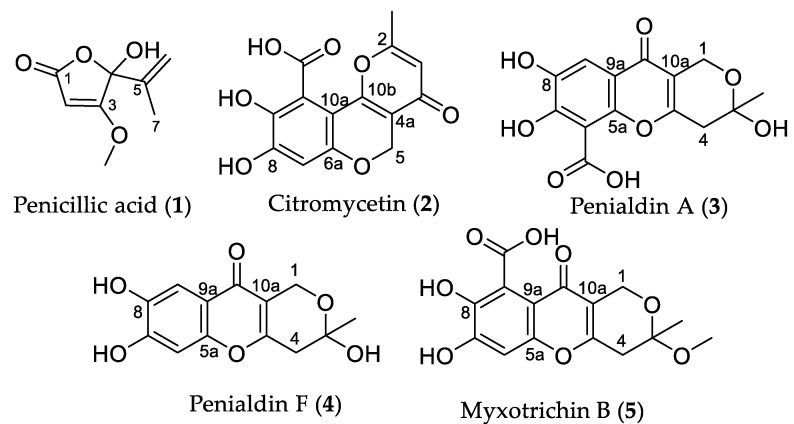
Structures of polyketide metabolites isolated from *Penicillium*.

The *Penicillium* genus is large with more than 350 currently accepted species divided into 28 sections [4]. Section *Aspergilloides* contains many common cosmopolitan species isolated from soil, food, and plants, including *Penicillium glabrum* ICMP 5686 isolated from sheep dung in New Zealand [5]. Section *Lanata-Divaricata* contains many common soil-inhabiting fungi, including *Penicillium bissetti* ICMP 21333 isolated from tree roots in New Zealand [6]. Investigation of ICMP 5685 and ICMP 21333 led to the isolation of five known polyketide secondary metabolites. Structure elucidation of secondary metabolites **1**–**5** with NMR spectroscopic and mass spectrometric data identified the natural products as penicillic acid (**1**) [7,8], citromycetin (**2**) [9], penialdin A (**3**) [10], penialdin F (**4**) [11], and myxotrichin B (**5**) [12] (Figure 1). The antimicrobial activities of **2**–**4** have been previously investigated and were found to be not active when tested against *S. aureus*, *E. coli*, *Bacillus subtilis*, and *Acinetobacter* sp. at concentrations less than 10 µg/mL [9,10,13]. However, **3** has been reported to exhibit anti-inflammatory activity [14], antioxidant activity [15], and moderate anti-diabetic activities [16]. Semisynthetic derivatization of penicillic acid (**1**), including hydrogenation and acetylation, resulted in the discovery of a novel keto acid derivative **1a** and two other known derivatives, **1b** and **1c**. The structure of keto acid derivative **1a** was suspected to be a hydrogenation product of the open chain tautomer of penicillic acid (**1**), the existence of which has somewhat been disagreed upon. Herein, we report the isolation, derivatization, structure elucidation, and bioactivities of polyketide secondary metabolites from *Penicillium* and provide evidence for the existence of the open-chain tautomer of penicillic acid (**1**).

## 2. Results and Discussion

### 2.1. Evaluation of Extract Bioactivity

**Figure 2:**
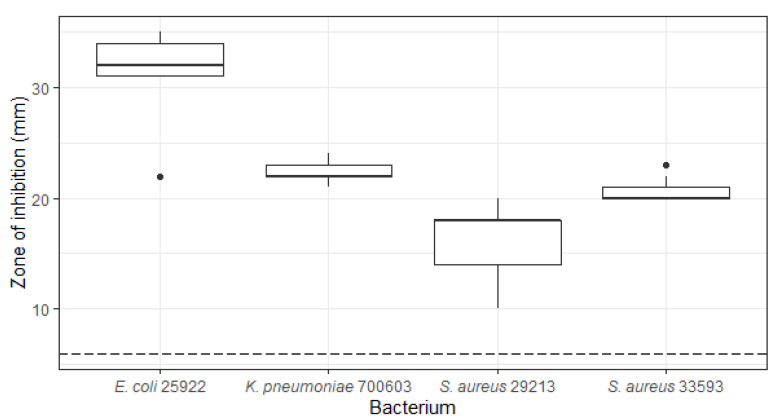
Antibacterial activity of *Penicillium bissettii* ICMP 21333 against *Escherichia coli*, *Klebsiella pneumoniae*, and *Staphylococcus aureus*. The antibacterial activity of *P. bissettii* ICMP 21333 was measured by the production of zones of inhibition (mm) when fungal plugs were incubated on lawns of *E. coli* ATCC 25922, *K. pneumoniae* ATCC 700603, *S. aureus* ATCC 29213 (antibiotic-sensitive), and *S. aureus* ATCC 33593 (antibiotic-resistant). Boxes are upper and lower quartiles with median shown. The whiskers extend up to 1.5× the inter-quartile range, and any dots beyond those bounds are outliers. The dotted line represents the diameter of the fungal plugs. Experiments were performed on three separate occasions with three technical replicates per experiment.

During initial whole cell screening, *Penicillium bissettii* (ICMP 21333) showed antibacterial activity against *E. coli*, *K. pneumoniae*, and antibiotic-sensitive and resistant *S. aureus*, with zones of inhibition of up to 35 mm in diameter (Figure 2).

From the crude extract of *Penicillium bissettii* ICMP 21333, a polyketide metabolite **1** was isolated via flash column chromatography. HRESI mass spectrometric data for **1** identified a sodiated adduct observed at *m/z* 193.0494 [M + Na]^+^ corresponding to a molecular formula of C_8_H_11_O_4_. The ^1^H NMR spectrum of **1** exhibited one broad exchangeable singlet (δ_H_ 7.87, OH), one sharp olefinic singlet (δ_H_ 5.41), two broad olefinic signals (δ_H_ 5.11 and 5.30), a methoxy singlet (δ_H_ 3.85), and a methyl singlet (δ_H_ 1.65). The ^13^C NMR spectrum showed eight signals at δ_C_ 179.4, 170.0, 140.2, 115.3, 102.4, 89.4, 59.8, and 17.1. HSQC NMR data connected the two olefinic signals (δ_H_ 5.11 and 5.30, H_2_-6) to a carbon signal at δ_C_ 115.3 (C-6), giving evidence for the presence of a terminal alkene. The HMBC spectrum identified correlations between these terminal alkene protons (δ_H_ 5.11 and 5.30, H_2_-6) and the methyl carbon at δ_C_ 17.1 (C-7) as well as the quaternary carbons at δ_C_ 102.4 (C-4) and 140.2 (C-5), suggesting the presence of a fragment with a methyl group adjacent to the terminal alkene. HMBC correlations between the other olefinic proton (δ_H_ 5.41, H-2) and both the carbonyl carbon at δ_C_ 179.4 (C-1) and quaternary carbon at δ_C_ 102.4 (C-4), and the correlation between the methoxy singlet (δ_H_ 3.85) and the other quaternary carbon at δ_C_ 170.0 (C-3), identified the presence of an α,β-unsaturated γ-lactone ring. Additional HMBC correlations between both the methyl (δ_H_ 1.65, H_3_-7) and olefinic (δ_H_ 5.41, H-2) protons and the quaternary carbon signal at δ_C_ 102.4 (C-4) connected the lactone ring to the terminal alkene fragment. The mass spectrometric and ^1^H NMR data were consistent with data previously reported for penicillic acid (**1**) (Figure 1) [7,8].

From the crude extracts of *P. glabrum*, four metabolites were isolated after purification by flash chromatography. Mass spectrometric analysis of metabolite **2** afforded a molecular formula of C_14_H_10_O_7_ from a sodiated adduct at *m/z* 313.0328 [M + Na]^+^. The ^1^H NMR spectrum showed the presence of four singlets corresponding to a methyl group (δ_H_ 2.35), a methylene group (δ_H_ 5.03), and two olefinic protons (δ_H_ 6.22 and 6.53). Analysis of the HMBC spectrum identified correlations between the olefinic proton at δ_H_ 6.53 (H-7) and four quaternary carbons at δ_C_ 104.7 (C-10a), 141.9 (C-9), 153.3 (C-8), and 158.1 (C-6a) and between the methylene protons at δ_H_ 5.03 (H_2_-5) and three quaternary carbons at δ_C_ 111.1 (C-4a), 152.5 (C-10b), and 158.1 (C-6a), indicating the presence of a chromene. The other olefinic proton at δ_H_ 6.22 (H-3) exhibited HMBC correlations to the methyl carbon at δ_C_ 19.1 (Me), a carbonyl carbon at δ_C_ 177.2 (C-4), and two quaternary carbons at δ_C_ 166.2 (C-2) and 111.1 (C-4a), indicating the presence of a γ-pyrone fused to the chromene ring. Substructure searching through the literature led to the identification of this metabolite as citromycetin (**2**) [9]. The other three metabolites showed similar ^1^H and ^13^C NMR spectra to **2**. Comparison of mass spectrometric and NMR data to the literature led to the identification of these metabolites as penialdin A (**3**) [10], penialdin F (**4**) [11], and myxotrichin B (**5**) [12].

### 2.2. Derivatisation of Penicillic Acid

**Figure 3:**
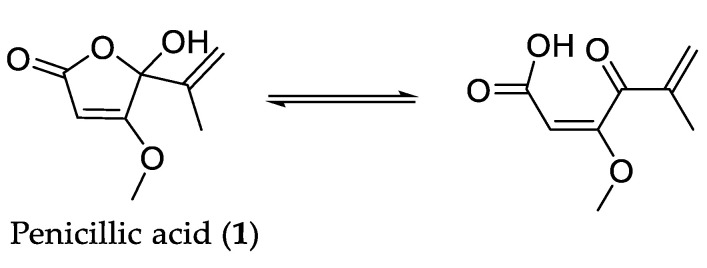
Tautomerization of penicillic acid (**1**).

Penicillic acid (**1**) was first reported in 1913 during an investigation between the incidence of pellagra and mould spoiled corn where they isolated an unknown fungal metabolite [17]. The structure of this metabolite was not determined until 1936 when Birkinshaw, Oxford, and Raistrick, who had been working on Alsberg and Black’s original cultures of *Penicillium puberculum*, were able to isolate the same compound in significantly higher yields from cultures of *P. cyclopium* [18]. They used this material to complete their investigation on the structure, which they reported as a substituted γ-keto acid that tautomerized to a γ-hydroxy lactone (Figure 3) [10].

There has been some disagreement on the existence of the open chain γ-keto acid tautomer [18,19,20,21]. The chemical studies carried out by Birkinshaw et al. provided evidence for the open-chain form [18]. They observed esterification of anhydrous penicillic acid (**1**) with diazomethane and the reaction of a cold aqueous solution of penicillic acid with one mole equivalent of free hydroxylamine, both of which they suggested provided evidence for the presence of the carboxyl group. However, a study by Raphael presented UV absorption spectra of both penicillic acid (**1**) and the methyl ester in acid and alkaline aqueous solutions in which a maximal absorbance was observed at ~220 nm for both compounds, which was consistent with the closed-ring lactone but not with the open-chain keto acid/keto ester [20]. Similar data for dihydropenicillic acid (**1b**), a derivative formed via hydrogenation of the natural product, were also obtained suggesting that penicillic acid exists purely as the lactone and that the keto acid tautomer is not required to explain the chemical properties observed by Birkinshaw et al. [18,20]. A study by Shaw also presented UV absorbance data for penicillic acid (**1**) in both acid and alkaline solutions [21]. Results comparable to Raphael’s were observed for the acid solution, but a shift in the absorption maxima to ~295 nm was observed in 0.1N NaOH solution, consistent with a change to the keto acid structure [20,21].

**Figure 4:**
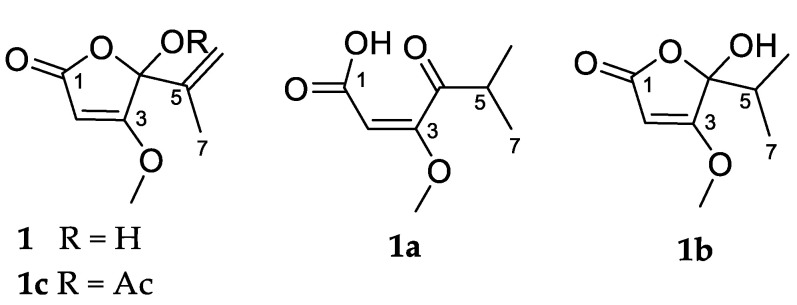
Structures of penicillic acid (**1**) and its derivatives (**1a**–**1c**).

To investigate the existence of the open chain γ-keto acid tautomer, in the present study, we proceeded to prepare dihydropenicillic acid (**1b**) (Figure 4) via catalytic hydrogenation of penicillic acid (**1**) in methanol, using palladium on carbon under a hydrogen atmosphere. The crude product was characterized via mass spectrometry and NMR. The sodiated adduct observed at *m/z* 195.0622 for [M + Na]^+^ in the (+)-HRESI mass spectrum of the crude product **1a** confirmed the addition of one mole of H_2_ to penicillic acid (**1**), giving a molecular formula of C_8_H_13_O_4_. The ^1^H NMR spectrum (Figure S4) of **1a** exhibited one sharp olefinic singlet (δ_H_ 5.19), one methoxy singlet (δ_H_ 3.72), and one dimethyl singlet (δ_H_ 1.07) (Table 1). The absence of the two olefinic signals of the terminal alkene in penicillic acid (**1**) and the presence of a dimethyl signal in the ^1^H NMR spectrum, suggested successful hydrogenation of the alkene at C-5/C-6. Of particular note was the absence of the expected septet signal of the H-5 proton and the expected splitting of the dimethyl signal into a doublet was also not observed. The HMBC NMR data of the crude product identified a correlation between the dimethyl singlet and a carbon signal at δc 39.3, consistent with an alkyl carbon at the C-5 position. Also present in the HMBC data was a correlation to a carbon signal at δc 217.1, consistent with the presence of a ketone, suggesting that **1a** was the ring opened keto acid structure as shown. This explained the absence of the ^1^H NMR signal for H-5 (α-keto proton) and the lack of splitting of the dimethyl signal, as it was speculated that the acidic α-keto proton had exchanged with deuterium from the CD_3_OD NMR solvent. This was confirmed by the ^1^H NMR spectrum recorded in non-exchangeable solvent CDCl_3_, which showed all expected signals: one sharp olefinic singlet (δ_H_ 5.12), one methoxy singlet (δ_H_ 3.82), one septet (δ_H_ 2.39, *J =* 7.0 Hz), and one dimethyl doublet (δ_H_ 1.01, *J =* 7.0 Hz) (Figure S6). Purification of **1a** was achieved using silica-gel-column chromatography. Changes were observed in the ^1^H NMR spectrum of the purified product (Figure S7) when compared to the crude product, necessitating further NMR characterization of the purified product **1b**.

The ^1^H NMR spectrum of the purified hydrogenation product **1b** showed one sharp olefinic singlet (δ_H_ 5.20), one methoxy singlet (δ_H_ 3.93), one septet (δ_H_ 2.12, *J =* 6.9 Hz), and two broad methyl doublets (δ_H_ 1.04, *J =* 6.8 Hz and δ_H_ 0.88, *J =* 6.8 Hz). The presence of the septet and the splitting of the methyl signals into doublets indicated that H-5 (δ_H_ 2.12, *J =* 6.9 Hz) of the purified product **1b** was not exchangeable with the NMR solvent, suggesting a change to the ring-closed lactone structure. The presence of two nonequivalent methyl signals was also consistent with the ring-closed form, with the chiral center at C-4 (δ_C_ 107.2) inducing non-equivalence in the pendant gem dimethyl groups. A change to the ring-closed structure was confirmed by HMBC data of the purified material, which exhibited a correlation between the two methyl proton signals and a carbon signal at δc 107.2 as well a correlation between the H-5 (δ_H_ 2.12, *J =* 6.9 Hz) proton signal and the same carbon signal at δc 107.2. The shift in this carbon signal was consistent with the shift observed for the hemiacetal carbon of penicillic acid (**1**) (δc 106.8). The HMBC data of the purified product also identified a second weaker set of correlations from a proton signal partially obscured by the broad methyl signal at δ_H_ 1.04, to carbons at δc 17.5, 39.3, and 217.1 (data not shown). This set of correlations suggested that some of the keto acid tautomer was present as the correlations were consistent with those observed in HMBC of the crude product. However, the expected second methoxy signal at δ_H_ 3.72 in the ^1^H NMR spectrum was absent. The NMR data for the crude and purified products (**1a** and **1b**) clearly show the existence of the two forms.

Acetylated penicillic acid (**1c**) was prepared via acetylation with acetic anhydride in pyridine over 18 h, under a nitrogen atmosphere. The crude material was purified by diol-bonded silica-gel-column chromatography. The sodiated adduct observed at *m/z* 235.0578 [M + Na]^+^ in the (+)-HRESI mass spectrum was consistent with the loss of a proton and the addition of an acetyl group, giving a molecular formula of C_10_H_12_O_5_. The ^1^H NMR spectrum (Figure S9) identified the introduction of new methyl protons (δ_H_ 2.12, s), which in turn exhibited a correlation in the HMBC spectrum to a carbon at δc 167.7, which is consistent with the introduction of an acetyl group. The mass spectrometric values and ^1^H NMR data obtained were in agreement with the literature [22].

### 2.3. ^13^C NMR Study of Penicillic Acid ***(1)*** Biosynthesis

**Figure 5:**
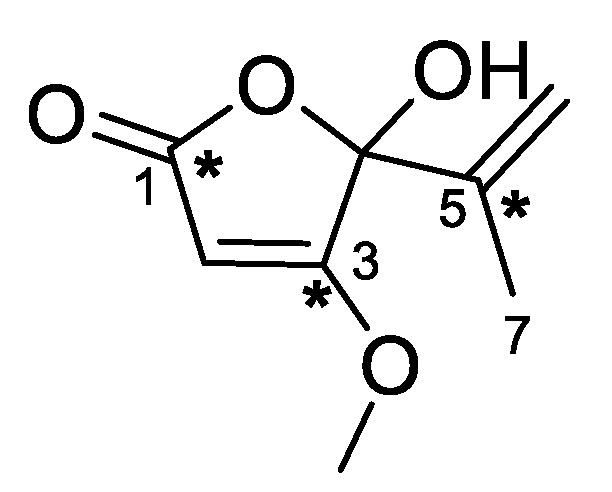
Labelling pattern observed in ^13^C NMR spectrum of penicillic acid (**1**) produced by cultures supplemented with 1-^13^C sodium acetate.

A sodium [1-^13^C] acetate labelling study was conducted to confirm the biosynthetic origin of penicillic acid (**1**). Cultures of *P. bissettii* ICMP 21333 were grown on PDA agar supplemented with 1g/L 1-^13^C sodium acetate. Extraction and fractionation as conducted previously afforded penicillic acid (**1**) (132 mg). The ^13^C NMR spectrum (Figure S3) of the ^13^C-labelled penicillic acid (**1**) was acquired and compared to the spectrum observed for non-labelled compound. The signals observed for C-1, C-3, and C-5 showed increased intensity, consistent with ^13^C enrichment. An indication of the extent of ^13^C enrichment was given by the relative peak integrals when compared to the spectrum of unlabeled penicillic acid (**1**). The relative peak integrals for labelled penicillic acid (**1**) clearly show enhancement for peaks at δc 181.2 (C-1, 12.3×), 173.2 (C-3, 10.1×), and 141.7 (C-5, 8.5×) (Table 2). The observed labelling pattern (Figure 5) was consistent with previous studies utilizing ^14^C-labelled acetate [23,24].

### 2.4. Bioactivity

Penicillic acid (**1**) and its derivatives **1b** and **1c**, as well as citromycetin (**2**) and penialdin A (**3**) and F (**4**), were screened for antimicrobial activity. Compounds **1**–**4** were tested in growth-inhibition assays against Methicillin-resistant *S. aureus* (MRSA), *E. coli*, *K. pneumoniae* (multi-drug resistant strain), *Acinetobacter baumannii, Pseudomonas aeruginosa, Candida albicans,* and *Cryptococcus neoformans* by the Community for Open Antimicrobial Drug Discovery (COADD) at The University of Queensland. The COADD test compound activity as an inhibition of microbial growth at a single concentration of 32 µg/mL is presented in Table 3.

At the concentration tested, penicillic acid (**1**) showed some inhibition of the growth of MRSA (53%) and *E. coli* (26%), as expected based on previous literature [25,26]. However, neither of the penicillic acid derivatives (**1b** and **1c**) showed any activity. Loss of activity on hydrogenation or acetylation of **1** suggests that the unsaturation at C-5 to C-6 is of importance as is the ability to convert to the open-chain form. Penialdin F (**4**) exhibited the strongest antimicrobial activity, inhibiting the growth of MRSA by over 71% (Table 3) at the concentration tested. This was surprising as **4** has been previously reported to be not active against wild-type and multidrug-resistant strains of *E. faecalis*, *E. faecium*, and *S. aureus* [13]. However, the inhibition was not complete, suggesting an MIC >32 µg/mL, which is considerably higher than antibiotics used for clinical treatment of antibiotic-sensitive *S. aureus* infections, which range from <0.001-~4 µg/mL [27] depending on the antibiotic. None of the other natural products showed strong enough inhibitory activity against any of the organisms, as expected. Citromycetin (**2**) has been previously investigated for antimicrobial activity (*E. coli*, *Bacillus subtilis*, and *Septoria nodurum*) and antiparasitic activity (*Haemonchus contortus*) and was found not active while also being non-cytotoxic against NS-1 cells [12]. Penialdin A (**3**) has also been reported as not active at a concentration of 10 µg/mL against *S. aureus*, *E. coli*, *B. subtilis*, and *Acinetobacter* sp. [10].

**Figure 6:**
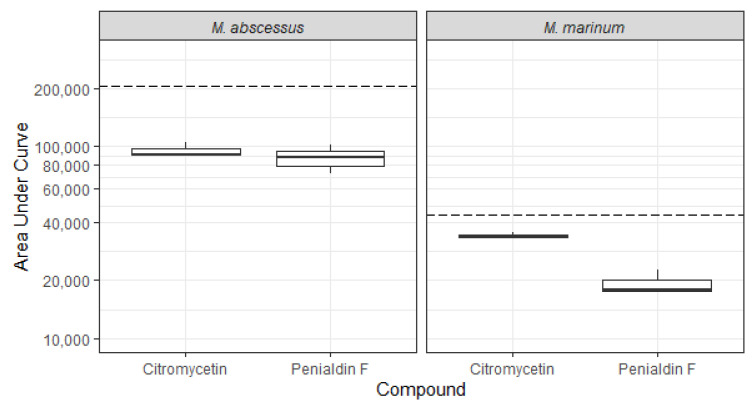
Effect of citromycetin (**2**) and penialdin F (**4**) on the luminescence of *Mycobacterium abscessus* BSG301 and *M. marinum* BSG101. The antibacterial activity of citromycetin (**2**) and penialdin F (**4**) were measured by changes in bacterial luminescence. Data are presented as box and whisker plots of the area under the curve values calculated from luminescence readings taken over 72 h. Boxes are upper and lower quartiles with median shown. The whiskers extend up to 1.5 × the inter-quartile range. The dotted line represents the no-compound control.

In addition to the assays performed by COADD, the compounds citromycetin (**2**) and penialdin F (**4**) were evaluated for their bioactivity against luminescent derivatives of *Mycobacterium abscessus* and *M. marinum* at a maximum concentration of 64 µg/mL using inhouse assays. Luminescence is frequently used as rapid readout of potential antimycobacterial activity [28,29,30]. Citromycetin and another compound of the penialdin family, penialdin C, have previously been shown to have activity against the Mycobacterial species *M. smegmatis*, with minimum inhibitory concentrations (MIC) of 31.2 and 15.6 μg/mL, respectively [31]. We found that citromycetin (**2**) and penialdin F (**4**) both displayed some activity against *M. abscessus* BSG301 and *M. marinum* BSG101, decreasing bacterial light output compared to the no-compound control (Figure 6). However, at the concentration tested, neither compound caused a reduction in light production of at least 1 log, which is how we define the MIC indicating their MIC against *M. abscessus* and *M. marinum* is greater than 64 μg/mL.

## 3. Materials and Methods

### 3.1. General Experimental Procedures

Infrared spectra were recorded on a Perkin–Elmer spectrometer 100 Fourier Transform infrared spectrometer equipped with a universal ATR accessory. Mass spectra were acquired on a Bruker micrOTOF Q II spectrometer (Bruker Daltonics, Bremen, Germany). Melting points were recorded on an electrothermal melting point apparatus and were uncorrected. ^1^H and ^13^C NMR spectra were recorded at 298K on a Bruker AVANCE 400 spectrometer (Bruker, Karlsruhe, Germany) at 400 and 100 MHz, respectively, using standard pulse sequences. Proto–deutero solvent signals were used as internal references (CD_3_OD: δ_H_ 3.31, δ_C_ 49.0; CDCl_3_: δ_H_ 0.00 (TMS), δ_C_ 77.16; DMSO-*d_6_*: δ_H_ 2.50, δ_C_ 39.52). For ^1^H NMR, the data are quoted as position (δ), relative integral, multiplicity (s = singlet, d = doublet, t = triplet, q = quartet, p = pentet, m = multiplet, dd = doublet of doublets, ddd = doublet of doublets of doublets, td = triplet of doublets, and br = broad), coupling constant (*J*, Hz), and assignment to the atom. The 13C NMR data are quoted as position (δ) and assignment to the atom. Flash column chromatography was carried out using Kieselgel 60 (40-63 μm) (Merck, Munich, Germany) silica gel or Merck Diol bonded silica (40–63 μm), with Merck C_8_ reversed–phase (40–63 μm) solid support. Gel filtration was carried out on Sephadex LH–20 (Pharmacia, Uppsala, Sweden). Thin-layer chromatography was conducted on Merck DC–plastikfolien Kieselgel 60 F254 plates. All solvents used were of analytical grade or better and/or purified according to standard procedures. Chemical reagents used were purchased from standard chemical suppliers and used as purchased.

### 3.2. Fungal Material

*P. bissettii* was described in 2016 from Canada [6] and later found to be present in South Korea [32] and New Zealand. The ecology of *P. bissettii* is unknown; however, it appears to be associated with tree soils or roots; the Canadian isolates were from Spruce forest soil and the Korean isolates from pine forest soil. Culture ICMP 21333 was isolated as an endophyte from the living roots of a kauri (*Agathis australis*) tree in Auckland, New Zealand in 2016. The identification of this isolate is supported by DNA sequences of the ITS (GENBANK# MW862794), BTUB (GENBANK# MZ393465), and CAL (GENBANK# MZ393460) genes. *P. glabrum* is a common cosmopolitan (GBIF 2021) filamentous ascomycete fungus. It has long been recognised as a cause of post-harvest fruit and vegetable rots since it was first described as *Citromyces glaber* in 1893. Culture ICMP 5686 was isolated from sheep dung in Palmerston North, New Zealand in July 1976. The identification of this isolate is supported by DNA sequences of the ITS (GENBANK# MW862782), BTUB (GENBANK# MZ393464), and CAL (GENBANK# MZ393462) genes. Cultures of *P. bissettii* and *P. glabrum* were grown at room temperature on Potato Dextrose Agar (PDA) (Fort Richard, New Zealand) plates. For extraction, the cultures were grown on 40 PDA plates until the fungus covered an area of 75% and were freeze dried for 5 days.

### 3.3. Antimicrobial Testing of Fungal Cultures

The ICMP fungal cultures were screened for antimicrobial activity after growth on PDA. Briefly, cultures of *E. coli* ATCC 25922, *K. pneumoniae* ATCC 700603, and *S. aureus* ATCC 29213 (antibiotic-sensitive (oxacillin)) and ATCC 33593 (antibiotic-resistant (gentamicin and methicillin)) were grown overnight in Tryptic Soy Broth (TSB) (Fort Richard, New Zealand) at 37 °C with shaking at 200 rpm. Cultures were diluted in TSB to an optical density at 600 nm of 0.1 and spread onto PDA plates for *E. coli* and *K. pneumoniae* and Mueller Hinton Agar (MHA) (Fort Richard, New Zealand) for *S. aureus* to produce a confluent bacterial lawn. Plugs were removed from fungal cultures using a 6 mm Paramount biopsy punch (Capes Medical, New Zealand) when the fungal cultures had grown to cover 75% of the plates. These were placed onto the inoculated PDA and MHA plates and incubated overnight at 37 °C. Any zones of inhibition were measured the next day using a ruler, and the diameter was recorded. Experiments were performed three times with three technical replicates per experiment.

### 3.4. Extraction and Isolation

Freeze-dried cultures of *P. bissettii* (17.49 g) were extracted with MeOH (1 L) for 4 h followed by CH_2_Cl_2_ (1 L) overnight. Concentration of the combined organic extracts afforded 2.46 g of crude extract. The crude extract was subjected to C_8_ flash column chromatography (H_2_O/MeOH), which afforded 5 fractions (A–E). Penicillic acid (**1**) was isolated from fraction C (1:1, H_2_O/MeOH) and further purified by Sephadex LH20 (MeOH) to give white crystals (25 mg).

The dry cultures of *P. glabrum* (20.86 g) were extracted with MeOH (1 L) for 4 h followed by CH_2_Cl_2_ (1 L) overnight. Concentration of the combined organic extracts afforded 2.03 g of crude extract. The crude extract was subjected to C_8_ flash column chromatography (H_2_O/MeOH), which afforded 5 fractions (A–E). Subsequent purification of fraction B (4:1, H_2_O/MeOH) by Sephadex LH20 (MeOH) afforded 5 fractions (B1–B5). Citromycetin (**2**) was isolated from fraction B4 after further purification by Diol-bonded silica column chromatography (*n*-Hexane/EtOAc, gradient) to give a yellow oil (2.9 mg). Purification of fraction C (1:1, H_2_O/MeOH) by Sephadex LH20 (MeOH) afforded 4 fractions (C1–C4). Penialdin A (**3**) (3.5 mg), penialdin F (**4**) (4.6 mg), and myxotrichin B (**5**) (0.75 mg) were isolated from fraction C2 after further purification by Diol-bonded silica column chromatography (*n*-Hexane/EtOAc, gradient).

#### 3.4.1. Penicillic acid (1)

^1^H NMR (CD_3_OD, 400 MHz) δ 5.41 (1H, br s, H-6), 5.27 (1H, s, H-2), 5.17 (1H, br s, H-6), 3.92 (3H, s, OMe), 1.75 (3H, dd, *J* = 1.0, 1.0 Hz, H_3_-7); ^13^C NMR (CD_3_OD, 100 MHz) 181.2 (C-1), 173.0 (C-3), 141.5 (C-5), 116.6 (C-6), 106.8 (C-4), 90.0 (C-2), 60.5 (C-8), 17.5 (C-7); ^1^H NMR (DMSO-*d*_6_, 400 MHz) δ 7.87 (1H, br s, OH), 5.41 (1H, s, H-2), 5.30 (1H, br s, H-6), 5.11 (1H, br s, H-6), 3.85 (3H, s, OMe), 1.65 (3H, s, H_3_-7); ^13^C NMR (DMSO-*d*_6_, 100 MHz) δ 179.4 (C-1), 170.0 (C-3), 140.2 (C-5), 115.3 (C-6), 102.4 (C-4), 89.4 (C-2), 59.8 (C-8), 17.1 (C-7); (+)-HRESIMS *m/z* 193.0474 [M + Na]^+^ (calcd for C_8_H_10_NaO_4_, 193.0470).

#### 3.4.2. Citromycetin (2)

^1^H NMR (CD_3_OD, 400 MHz) δ 6.53 (1H, s, H-7), 6.22 (1H, s, H-3), 5.03 (2H, s, H_2_-5), 2.35 (3H, s, Me); ^13^C NMR (CD_3_OD, 100 MHz) δ 177.2 (C-4), 167.0 (COOH), 166.1 (C-2), 158.1 (C-6a), 153.3 (C-8), 152.5 (C-10b), 141.9 (C-9), 120.0 (C-10), 113.3 (C-3), 111.1 (C-4a), 104.7 (C-10a), 104.5 (C-6a), 62.7 (C-5), 19.1 (Me); (+)-HRESIMS *m/z* 313.0328 [M + Na]^+^ (calcd for C_14_H_10_NaO_7_, 313.0319).

#### 3.4.3. Penialdin A (3)

^1^H NMR (CD_3_OD, 400 MHz) δ 7.09 (1H, s, H-9), 4.65 (2H, s, H_2_-1), 2.99 (1H, d, *J* = 17.8 Hz, H_2_-4_A_), 2.77 (1H, d, *J* = 17.8 Hz, H_2_-4_B_), 1.57 (3H, s, Me); ^13^C NMR (CD_3_OD, 100 MHz) δ 175.2 (C-10), 168.0 (COOH), 160.8 (C-4a), 154.8 (C-8), 152.6 (C-5a), 144.1 (C-7), 119.1 (C-6), 114.6 (C-10a), 111.8 (C-9a), 105.6 (C-9), 94.3 (C-3), 56.1 (C-1), 37.2 (C-5), 27.2 (Me); (+)-HRESIMS *m/z* 287.0529 [M + Na]^+^ (calcd for C_13_H_12_NaO_6_, 287.0526).

#### 3.4.4. Penialdin F (4)

^1^H NMR (CD_3_OD, 400 MHz) δ 7.36 (1H, s, H-9), 6.86 (1H, s, H-6), 4.62 (2H, s, H_2_-1), 2.91 (1H, d, *J* = 17.0 Hz, H_2_-4_A_), 2.68 (1H, d, *J* = 17.0 Hz, H_2_-4_B_), 1.55 (3H, s, Me); ^13^C NMR (CD_3_OD, 100 MHz) δ 174.9 (C-10), 160.8 (C-4a), 151.9 (C-5a, C-8), 144.3 (C-7), 115.7 (C-9a), 114.6 (C-10a), 106.7 (C-9), 101.9 (C-6), 94.3 (C-3), 46.1 (C-1), 37.1 (C-5), 27.2 (Me); (+)-HRESIMS *m/z* 331.0428 [M + Na]^+^ (calcd for C_14_H_12_NaO_8_, 331.0424).

#### 3.4.5. Myxotrichin B (5)

^1^H NMR (CD_3_OD, 400 MHz) δ 7.09 (1H, s, H-6), 4.68 (1H, d, *J* = 15.0 Hz, H_2_-1_A_), 4.40 (1H, d, *J* = 15.0 Hz, H_2_-1_B_), 3.33 (3H, s, OMe), 3.02 (1H, d, *J* = 17.9 Hz, H_2_-4_A_), 2.81 (1H, d, *J* = 17.9 Hz, H_2_-4_B_), 1.54 (3H, s, Me); ^13^C NMR (CD_3_OD, 100 MHz) δ 175.5 (C-10), 167.8 (COOH), 160.4 (C-4a), 154.1 (C-7), 151.5 (C-5a), 144.4 (C-7), 118.4 (C-9), 113.8 (C-10a), 111.8 (C-9a), 105.1 (C-6), 98.2 (C-3), 57.0 (C-1), 48.3 (OMe), 37.2 (C-5), 21.3 (Me); (+)-HRESIMS *m/z* 345.0588 [M + Na]^+^ (calcd for C_15_H_14_NaO_8_, 345.0581).

#### 3.4.6. (E)-3-Methoxy-5-methyl-4-oxohex-2-enoic acid (1a)

To a solution of penicillic acid (**1**) (5 mg, 0.03 mmol) in anhydrous MeOH (1 mL) was added Pd/C (1 mg, 0.003 mmol), and the reaction was stirred under an H_2_ atmosphere for 18 h. The reaction mixture was filtered through celite and washed with MeOH (50 mL). The solvent was removed under reduced pressure to give the crude product as a pale-yellow oil (3.43 mg, 66%).

^1^H NMR (CD_3_OD, 400 MHz) δ 5.19 (1H, s, H-2), 3.72 (3H, s, OMe), 3.31 (1H, obs by solvent, H-5), 1.07 (6H, s, H_3_-6, H_3_-7); ^13^C NMR (CD_3_OD, 100 MHz) δ 217.2 (C-4), 174.6 (C-1), 171.2 (C-3), 97.2 (C-2), 57.8 (OMe), 39.2 (C-5), 17.7 (C-6, C-7); ^1^H NMR (CDCl_3_, 400 MHz) δ 5.12 (1H, s, H-2), 3.82 (3H, s, OMe), 2.39 (1H, septet, *J =* 7.0 Hz, H-5) 1.01 (6H, d, *J =* 7.0 Hz, H_3_-6, H_3_-7); (+)-HRESIMS *m/z* 195.0622 [M + Na]^+^ (calcd for C_8_H_12_NaO_4_, 195.0630).

#### 3.4.7. 5-Hydroxy-5-isopropyl-4-methoxyfuran-2(5H)-one (1b)

The product was purified by silica-gel column chromatography (10% MeOH:CH_2_Cl_2_) to give the product as white crystals (1.72 mg, 33%).

^1^H NMR (CD_3_OD, 400 MHz) δ 5.20 (1H, s, H-2), 3.93 (3H, s, OMe), 2.12 (1H, septet, *J =* 6.8 Hz, H-5), 1.04 (3H, d, *J =* 6.4 Hz H_3_-6), 0.88 (3H, d, *J =* 6.4 Hz, H_3_-7); ^13^C NMR (CD_3_OD, 100 MHz) δ 182.1 (C-3), 173.5 (C-1), 107.2 (C-4), 89.2 (C-2), 59.9 (OMe), 34.6 (C-5), 16.4 (C-7), 16.1 (C-6); ^1^H NMR (CDCl_3_, 400 MHz) δ 5.06 (1H, s, H-2), 3.91 (3H, s, OMe), 2.21 (1H, p, *J =* 6.8 Hz, H-5), 1.12–1.02 (3H, m, H_3_-6), 0.96–0.88 (3H, m, H_3_-7).

#### 3.4.8. 3-Methoxy-5-oxo-2-(prop-1-en-2-yl)-2,5-dihydrofuran-2-yl acetate (1c)

To a solution of penicillic acid (**1**) (5 mg, 0.03 mmol) in pyridine (1 mL) was added acetic anhydride (0.05 mL, 0.52 mmol) dropwise, and the reaction was stirred for 18 hr under a nitrogen atmosphere. The reaction mixture was quenched with 10% HCl and extracted with CH_2_Cl_2_ (3 × 10 mL) and ethyl acetate (1 × 10 mL). The combined organic layers were dried with MgSO_4_ and the solvent removed under reduced pressure. The product was purified by diol-bonded silica-gel column chromatography, eluting with CH_2_Cl_2_, to give the product as a brown oil (1.96 mg, 30%).

^1^H NMR (CDCl_3_, 400 MHz) δ 5.38 (1H, br s, H-6), 5.17 (2H, br s, H-2, H-6), 3.91 (3H, s, OMe), 2.12 (3H, s, OAc), 1.83 (3H, d, *J* = 1.0 Hz, H_3_-7); ^13^C NMR (CDCl_3_, 100 MHz) δ 178.1 (C-1), 168.9 (C-3), 167.7 (OAc), 138.4 (C-5), 116.1 (C-6), 101.6 (C-4), 89.8 (C-2), 59.8 (OMe), 21.3 (OAc), 17.3 (C-7); (+)-HRESIMS *m/z* 235.0578 [M + Na]^+^ (calcd for C_10_H_12_NaO_5_, 235.0580).

### 3.5. Sodium [1-^13^C] Acetate Incorporated Acetate Fermentation of P. bissettii

#### Extraction and Isolation

Cultures of *Penicillium bissettii* were grown on 17 PDA plates supplemented with 1g/L 1-^13^C sodium acetate until the fungus covered 75% of the plate. The cultured plates were freeze dried (11.00 g) and extracted with methanol and dichloromethane to give 2.28 g of crude extract. The crude extract was purified via C_8_ reverse-phased column chromatography (H_2_O/MeOH), and penicillic acid (**1**) (132 mg) was isolated from fraction C (1:1, H_2_O/MeOH).

### 3.6. Antimicrobial Assays of Pure Compounds

Antimicrobial evaluation of the pure compounds against *S. aureus* ATCC 43300 (MRSA), *E. coli* ATCC 25922, *Pseudomonas aeruginosa* ATCC 27853, *Klebsiella pneumoniae* ATCC 700603, *Acinetobacter baumannii* ATCC 19606, *Candida albicans* ATCC 90028, and *Cryptococcus neoformans* ATCC 208821 was undertaken at the Community for Open Antimicrobial Drug Discovery at The University of Queensland (Queensland, Australia) according to their standard protocols [33]. For antimicrobial assays, the tested strains were cultured in either Luria broth (LB) (In Vitro Technologies, USB75852, Victoria, Australia), nutrient broth (NB) (Becton Dickson 234000, New South Wales, Australia), or MHB at 37 °C overnight. A sample of culture was then diluted 40-fold in fresh MHB and incubated at 37 °C for 1.5−2 h. The compounds were serially diluted 2-fold across the wells of 96-well plates (Corning 3641, nonbinding surface), with compound concentrations ranging from 0.015 to 64 μg/mL, plated in duplicate. The resultant mid-log-phase cultures were diluted to the final concentration of 1 × 10^6^ CFU/mL; then, 50 μL were added to each well of the compound containing plates giving a final compound concentration range of 0.008–32 μg/mL and a cell density of 5 × 10^5^ CFU/mL. All plates were then covered and incubated at 37 °C for 18 h. Inhibition of bacterial growth was determined measuring absorbance at 600 nm (OD600), using a Tecan M1000 Pro monochromator plate reader. The percentage of growth inhibition was calculated for each well, using the negative control (media only) and positive control (bacteria without inhibitors) on the same plate as references.

For the antifungal assay, fungi strains were cultured for 3 days on Yeast Extract Peptone Dextrose (YPD) agar at 30 °C. A yeast suspension of 1 × 10^6^ to 5 × 10^6^ CFU/mL was prepared from five colonies. These stock suspensions were diluted with yeast nitrogen base (YNB) (Becton Dickinson, 233520, New South Wales, Australia) broth to a final concentration of 2.5 × 10^3^ CFU/mL. The compounds were serially diluted 2-fold across the wells of 96-well plates (Corning 3641, nonbinding surface), with compound concentrations ranging from 0.015 to 64 μg/mL and final volumes of 50 μL, plated in duplicate. Then, 50 μL of the fungi suspension that was previously prepared in YNB broth to the final concentration of 2.5 × 10^3^ CFU/mL were added to each well of the compound-containing plates, giving a final compound concentration range of 0.008–32 μg/mL. Plates were covered and incubated at 35 °C for 36 h without shaking. *C. albicans* MICs were determined by measuring the optical density at 530 nm. For *C. neoformans*, resazurin was added at 0.006% final concentration to each well and incubated for a further 3 h before MICs were determined by measuring the optical density at 570–600 nm.

Colistin and vancomycin were used as positive bacterial inhibitor standards for Gram-negative and Gram-positive bacteria, respectively. Fluconazole was used as a positive fungal inhibitor standard for *C. albicans* and *C. neoformans*. The antibiotics were provided in 4 concentrations, with 2 above and 2 below its MIC value, and they were plated into the first 8 wells of column 23 of the 384-well NBS plates. The quality control (QC) of the assays was determined by the antimicrobial controls and the Z’-factor (using positive and negative controls). Each plate was deemed to fulfil the quality criteria (pass QC) if the Z’-factor was above 0.4, and the antimicrobial standards showed a full range of activity, with full growth inhibition at their highest concentration and no growth inhibition at their lowest concentration.

Antimicrobial evaluation against *M. abscessus* and *M. marinum* was undertaken using inhouse assays. *M. abscessus* BSG301 and *M. marinum* BSG101 [28] are stable bioluminescent derivatives transformed with the integrating plasmid pMV306G13ABCDE [34]. This allows light production to be used as a surrogate for bacterial viability [28,29,30]. Mycobacterial cultures were grown shaking at 200 rpm in Middlebrook 7H9 broth (Fort Richard, Auckland, New Zealand) supplemented with 10% Middlebrook ADC enrichment media (Fort Richard, Auckland, New Zealand), 0.4% glycerol (Sigma-Aldrich, St. Louis, MO, USA), and 0.05% tyloxapol (Sigma-Aldrich, St. Louis, MO, USA). *M. abscessus* was grown at 37 °C and *M. marinum* at 28 °C. Cultures were grown until they reached the stationary phase (approximately 3–5 days for *M. abscessus* BSG301 and 7–10 days for *M. marinum* BSG101) and then diluted in Mueller Hinton broth II (MHB) (Fort Richard, Auckland, NewZealand) supplemented with 10% Middlebrook ADC enrichment media and 0.05% tyloxapol to give an optical density at 600 nm (OD600) of 0.001, which is the equivalent of ~10^6^ bacteria per mL. Pure compounds were dissolved in DMSO and added in duplicate to the wells of a black 96-well plate (Nunc, Thermo Scientific, Waltham, MA, USA) at doubling dilutions with a maximum concentration of 128 μg/mL. Then, 50 μL of diluted bacterial culture was added to each well of the compound containing plates giving final compound concentrations of 0–64 μg/mL and a cell density of ~5 × 10^5^ CFU/mL. Rifampicin (Sigma-Aldrich, St. Louis, MO, USA) was used as positive control at 1000 μg/mL for *M. abscessus* and 10 μg/mL for *M. marinum*. Between measurements, plates were covered, placed in a plastic box lined with damp paper towels, and incubated with shaking at 100 rpm at 37 °C for *M. abscessus* and 28 °C for *M. marinum*. Bacterial luminescence was measured at regular intervals over 72 h using a Victor X-3 luminescence plate reader (PerkinElmer, Boston, MA, USA) with an integration time of 1 s. We defined the MIC as causing a 1-log reduction in light production, as previously described [29]. More detailed protocols are available at protocols.io (accessed on 19 November 2021) [35,36].

### 3.7. Statistical Analysis

Statistical analysis was performed as described using GraphPad Prism version 8.4.3.

## 4. Conclusions

Investigation of the secondary metabolites of two strains of *Penicillium* led to the isolation of five known natural products. Antimicrobial screening of selected natural products identified penicillic acid (**1**) and penialdin F (**4**), which exhibited good activity against MRSA with the latter also exhibiting weak antimycobacterial activity against *M. abscessus* and *M. marinum* along with citromycetin (**2**). To the best of our knowledge, this is the first instance where these compounds have been evaluated for antimycobacterial activity. Derivatisation of **1** through hydrogenation led to the synthesis of a novel dihydro (**1a**) derivative, which provided evidence to the existence of an open-chained γ-keto acid tautomer, with purification attempts leading to ring closure of **1a**, forming the lactone **1b**. Although none of the natural products or their derivatives exhibited significant antimicrobial activity to be of interest as lead compounds for antibiotic development, the ICMP collection remains a vast untapped resource for novel bioactive natural products worthy of further exploration.

## Figures and Tables

**Table 1:** ^1^H and ^13^C NMR (CD_3_OD) signals observed for crude (**1a**) and purified (**1b**) products.

Position	1a		1b	
	δ_H_	δ_C_	δ_H_	δ_C_
1		174.6		173.5
2	5.19	97.2	5.20	89.2
3		171.2		182.1
4		217.1		107.2
5	Not observed	39.3	2.12	34.6
6	1.07	17.5	1.04	16.1
6′	1.07	17.5	0.88	16.4
7	3.72	57.8	3.93	59.9

**Table 2:** Relative Integral Values for ^13^C NMR Peaks Observed for ^13^C-labelled Penicillic Acid (**1**).

Position	Δc	Relative Integral (Labelled/Unlabelled)
C-1	181.2	12.3
C-2	90.1	1.0
C-3	173.2	10.1
C-4	Not observed	
C-5	141.7	8.5
C-6	116.7	1.1
C-7	17.5	1.0
C-8	60.4	0.9

**Table 3:** Antimicrobial and antifungal activities of natural products **1**–**4** and derivatives **1b** and **1c**.

Compound	Percentage Inhibition at 32 µg/mL						
	*S. a* ^a^	*E. c* ^b^	*K. p* ^c^	*P. a* ^d^	*A. b* ^e^	*C. a* ^f^	*C. n* ^g^
**1**	53.07 ****	26.11 ***	18.06 *	−1.15	6.12	6.50	2.15
**1b**	−1.05	0.34	4.32	2.75	−26.55	2.49	18.33 **
**1c**	17.41 *	5.15	9.34	−10.06	−11.88	2.56	−7.04
**2**	14.22	11.16	13.49	4.70	−4.31	−1.86	1.11
**3**	−7.23	13.60	−7.65	−4.08	−24.24	−2.20	20.72 **
**4**	71.52 ****	9.29	15.62 *	0.52	12.55	8.84	6.73

All values are presented as the mean (*n* = 2). Negative values represent increases in growth compared to the no-compound controls. Data were analysed using a two-way ANOVA with Dunn’s multiple comparison test. The adjusted p values of those compounds that were significantly different to the relevant no inhibition control are given by * (* *p* < 0.05; ** *p* < 0.005; *** *p* < 0.0005; **** *p* < 0.0001). ^a^ *Staphylococcus aureus* ATCC 43300 (MRSA) with vancomycin (MIC 1 μg/mL) used as a positive control; ^b^ *Escherichia coli* ATCC 25922 with colistin (MIC 0.125 μg/mL) used as a positive control; ^c^ *Klebsiella pneumoniae* ATCC 700603 with colistin (MIC 0.25 μg/mL) as a positive control; ^d^ *Pseudomonas aeruginosa* ATCC 27853 with colistin (MIC 0.25 μg/mL); ^e^ *Acinetobacter baumanii* ATCC 19606 with colistin (MIC 0.25 μg/mL) as a positive control; ^f^ *Candida albicans* ATCC 90028 with fluconazole (MIC 0.125 μg/mL) as a positive control; ^g^ *Cryptococcus neoformans* ATCC 208821 with fluconazole (MIC 8 μg/mL) as a positive control.

## Supplementary Materials

The following are available online, Figures S1–S10: ^1^H and ^13^C NMR spectra of **1**, **1a,** and **1c** and the ^1^H NMR spectrum of **1b** as well as the ^13^C NMR spectrum of 1-^13^C labelled **1**.

## Sample Availability

Samples of compounds **1**–**4** are available from the authors.
