# Supplementary material for: Antimicrobial Polyketide Metabolites from Penicillium bissettii and P. glabrum
Source: Molecules. 2021 Dec 31;27(1):240. doi: 10.3390/molecules27010240 (PMC8746583; doi:10.3390/molecules27010240)
Supplement: Supplementary file 1 [file molecules-27-00240-s001.zip › molecules-1500517-Supplementary.pdf]

Supporting Information

Antimicrobial polyketide metabolites from *Penicillium bissettii* and *P. glabrum.*

Melissa M. Cadelis[1,2,*], Natasha S. L. Nipper[1], Alex Grey[2], Soeren Geese[2], Shara J. van de Pas[2], Bevan S. Weir[3], Brent R. Copp[1,†] and Siouxsie Wiles[2,*,†]

[1]School of Chemical Sciences, University of Auckland - Waipapa Taumata Rau, Private Bag 92019, Auckland 1142, Aotearoa New Zealand
[2]Bioluminescent Superbugs Lab, School of Medical Sciences, University of Auckland - Waipapa Taumata Rau, Private Bag, 92019, Auckland 1142, Aotearoa New Zealand
[3]Manaaki Whenua - Landcare Research, Private Bag 92170, Auckland 1142, Aotearoa New Zealand

*Corresponding Author: m.cadelis@auckland.ac.nz and s.wiles@auckland.ac.nz
†These authors contributed equally to this work.

Contents

Figure S1. ^{1}H NMR spectrum (400 MHz, CD_3OD) of **1** **2**
Figure S2. ^{13}C NMR spectrum (100 MHz, CD_3OD) of **1** **3**
Figure S3. ^{13}C NMR spectrum (100 MHz, CD_3OD) of 1-^{13}C acetate labelled **1** **4**
Figure S4. ^{1}H NMR spectrum (400 MHz, CD_3OD) of **1a** **5**
Figure S5. ^{13}C NMR spectrum (100 MHz, CD_3OD) of **1a** **6**
Figure S6. ^{1}H NMR spectrum (400 MHz, $CDCl_3$) of **1a** **7**
Figure S7. ^{1}H NMR spectrum (400 MHz, CD_3OD) of **1b** **8**
Figure S8. ^{1}H NMR spectrum (400 MHz, $CDCl_3$) of **1b** **9**
Figure S9. ^{1}H NMR spectrum (400 MHz, $CDCl_3$) of **1c** **10**
Figure S10. ^{13}C NMR spectrum (100 MHz, $CDCl_3$) of **1c** **11**

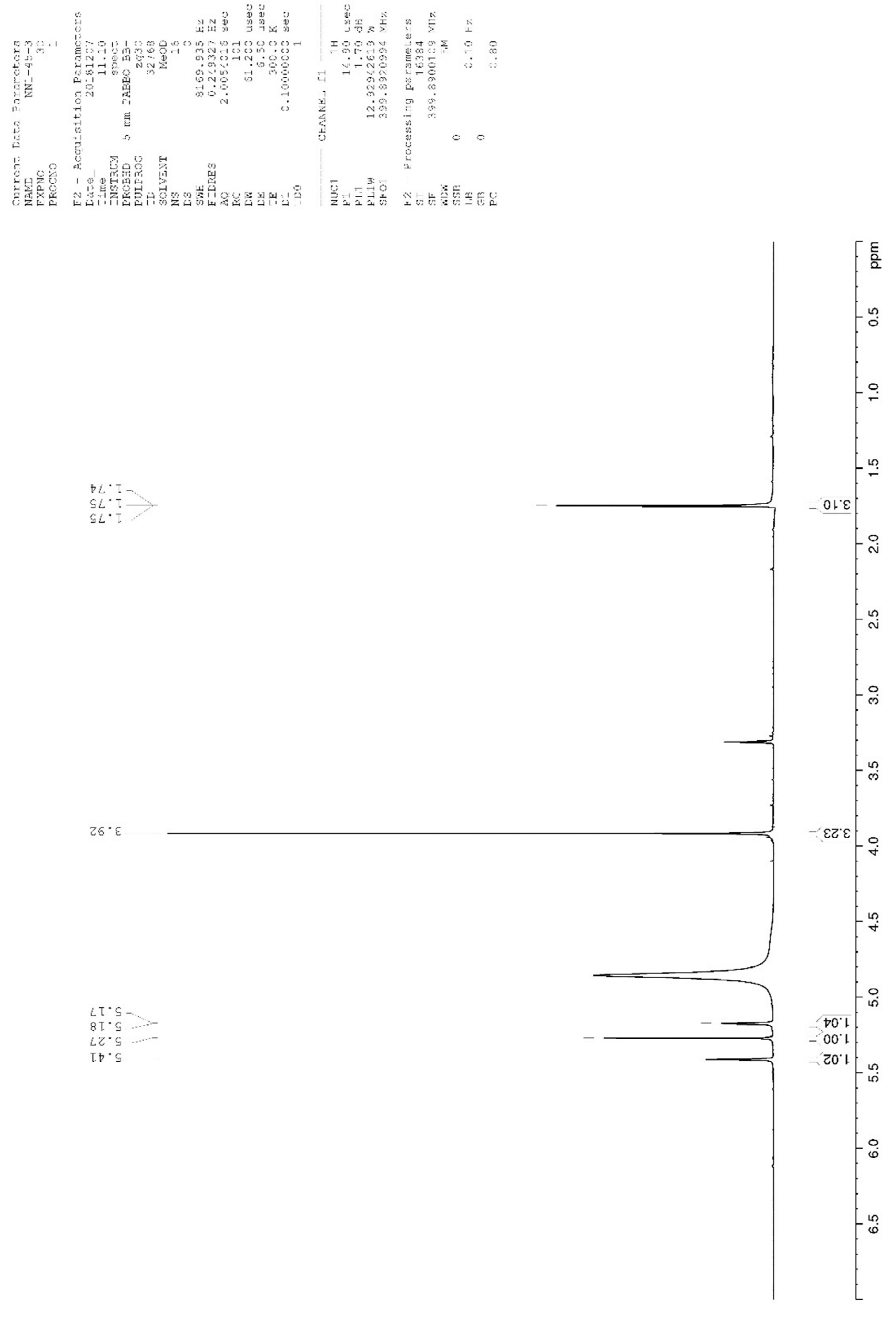

Figure S1. ^{1}H NMR spectrum (400 MHz, CD_3OD) of **1**

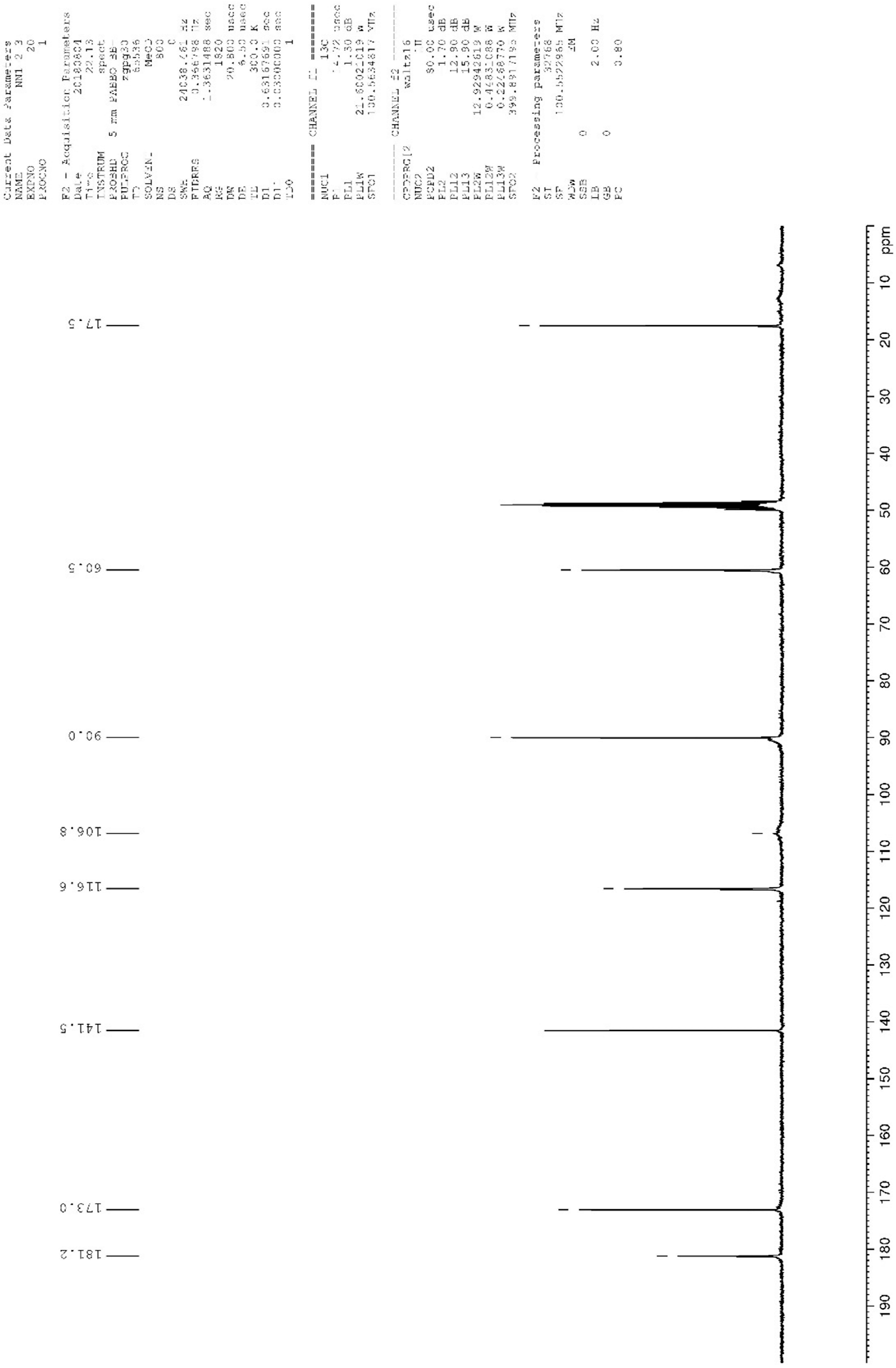

Figure S2. ^{13}C NMR spectrum (100 MHz, CD_3OD) of **1**

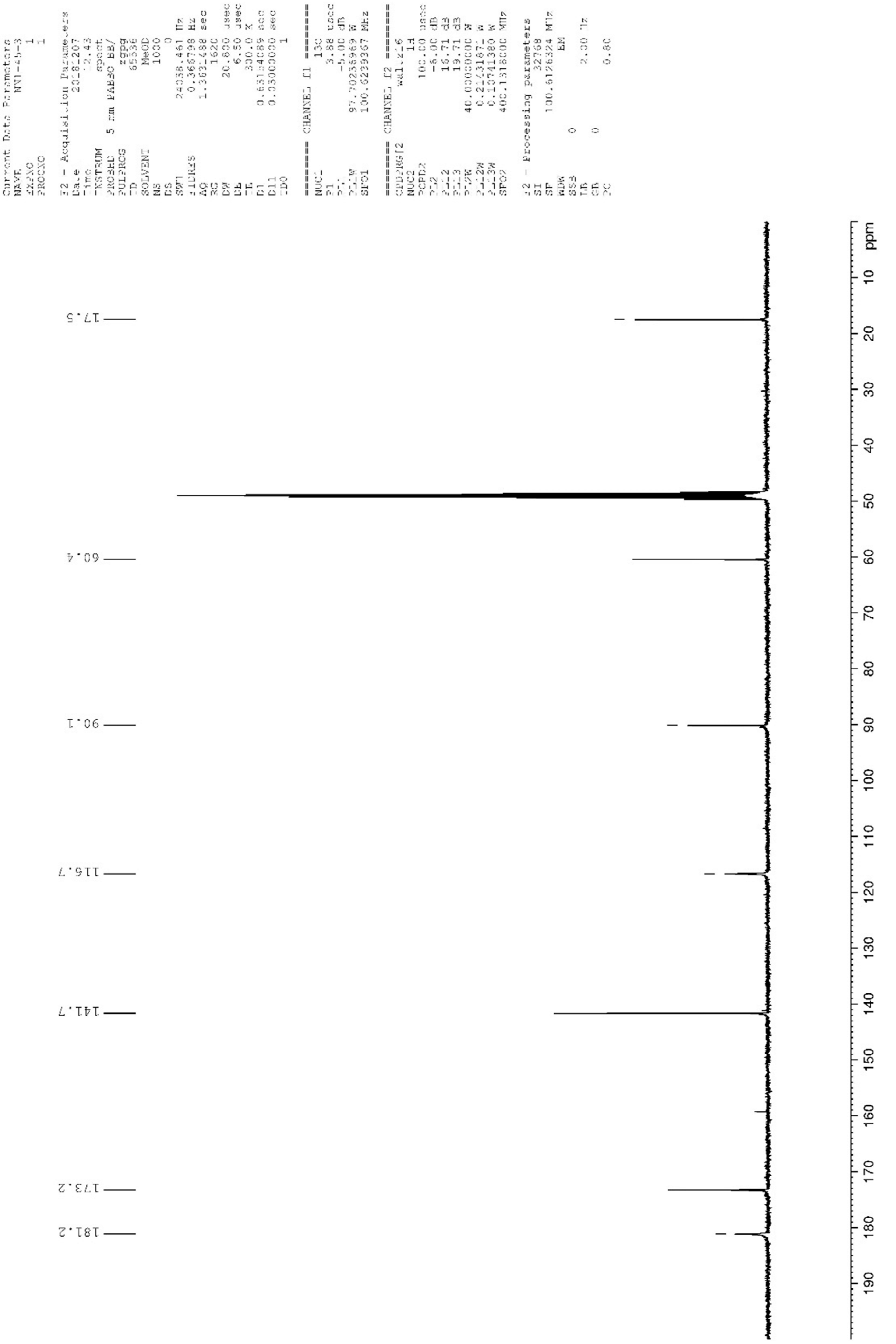

Figure S3. ^{13}C NMR spectrum (100 MHz, CD_3OD) of 1-^{13}C acetate labelled **1**

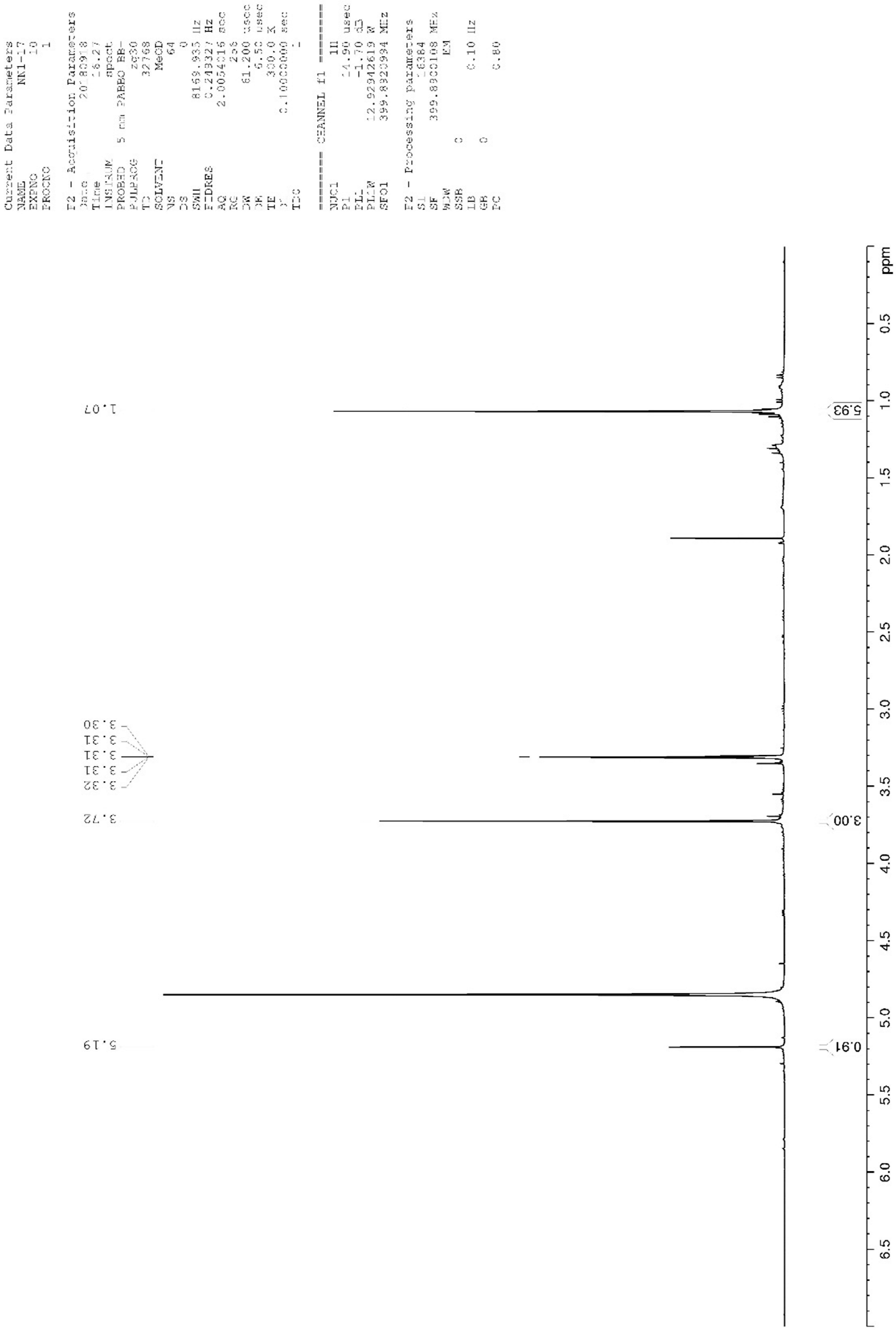

Figure S4. ^{1}H NMR spectrum (400 MHz, CD_3OD) of **1a**

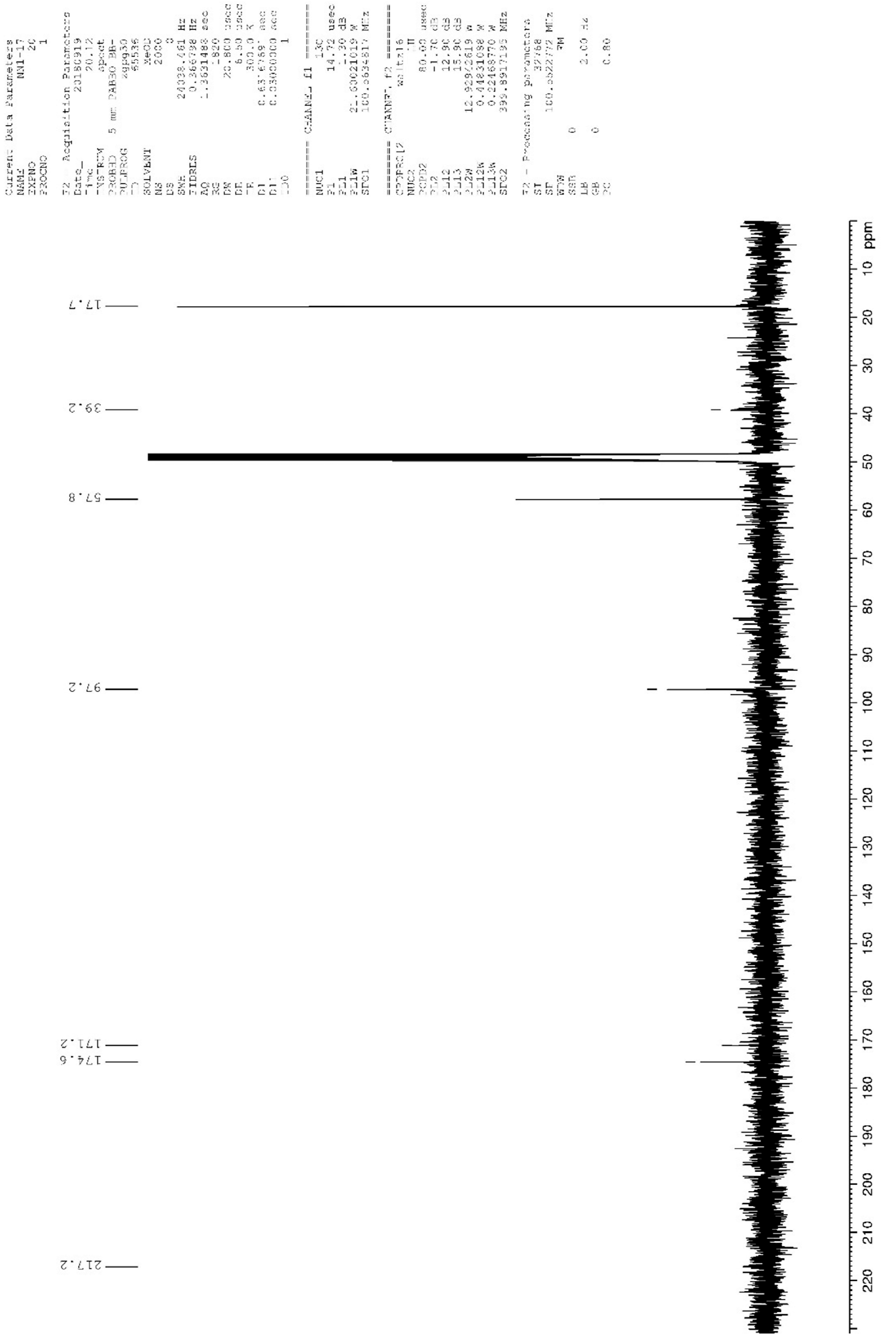

Figure S5. ^{13}C NMR spectrum (100 MHz, CD_3OD) of **1a**

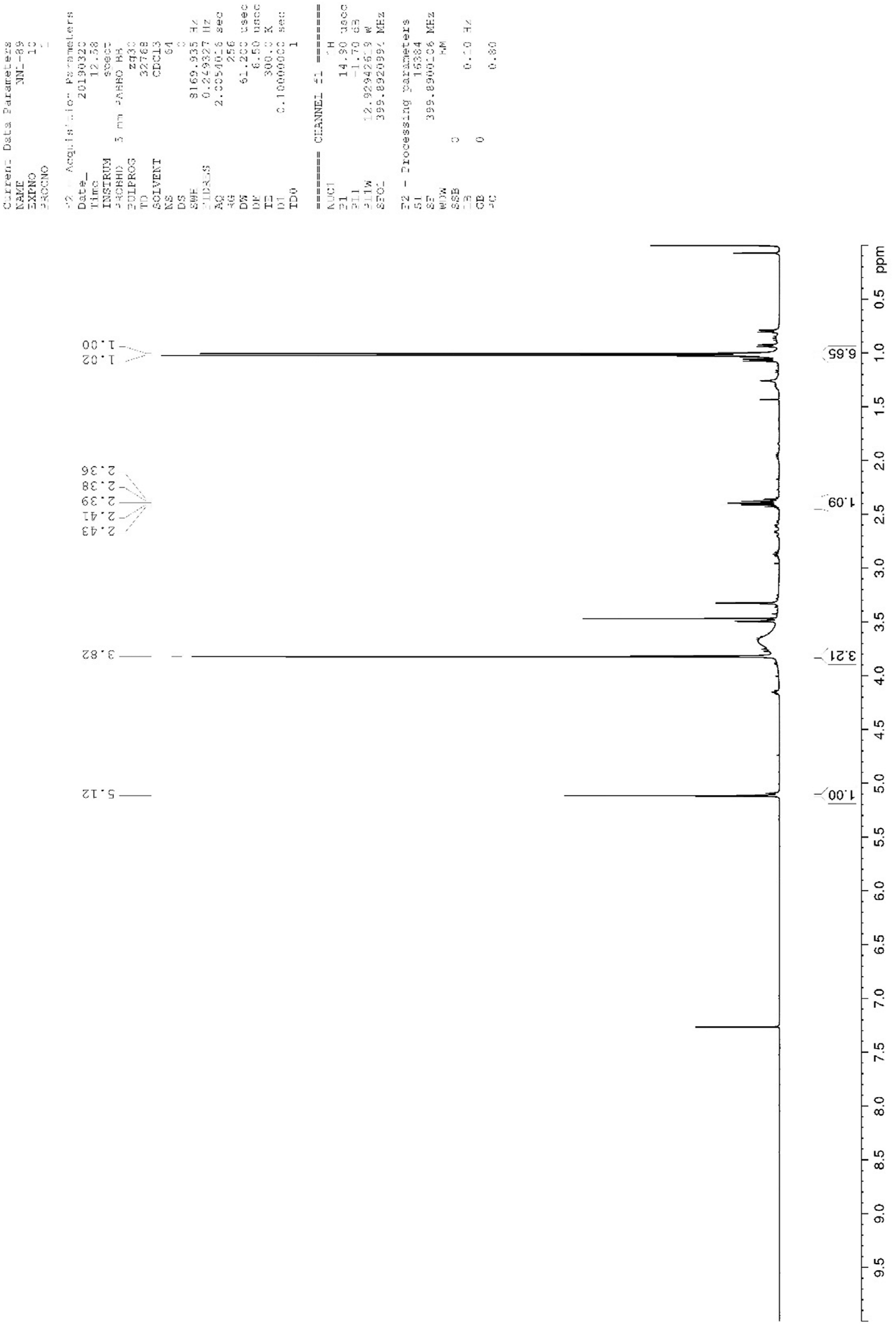

Figure S6. ^{1}H NMR spectrum (400 MHz, $CDCl_3$) of **1a**

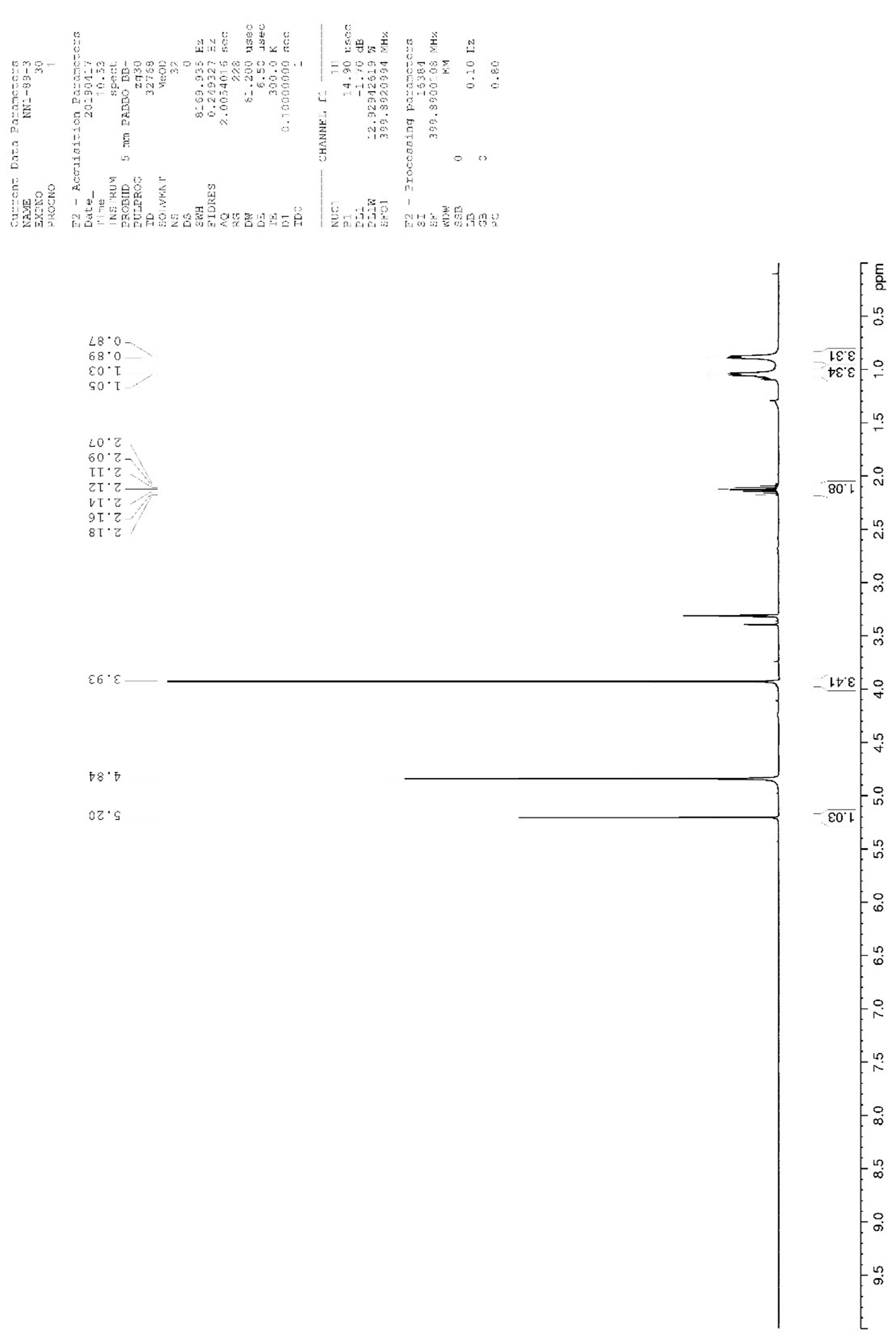

Figure S7. ^{1}H NMR spectrum (400 MHz, CD_3OD) of **1b**

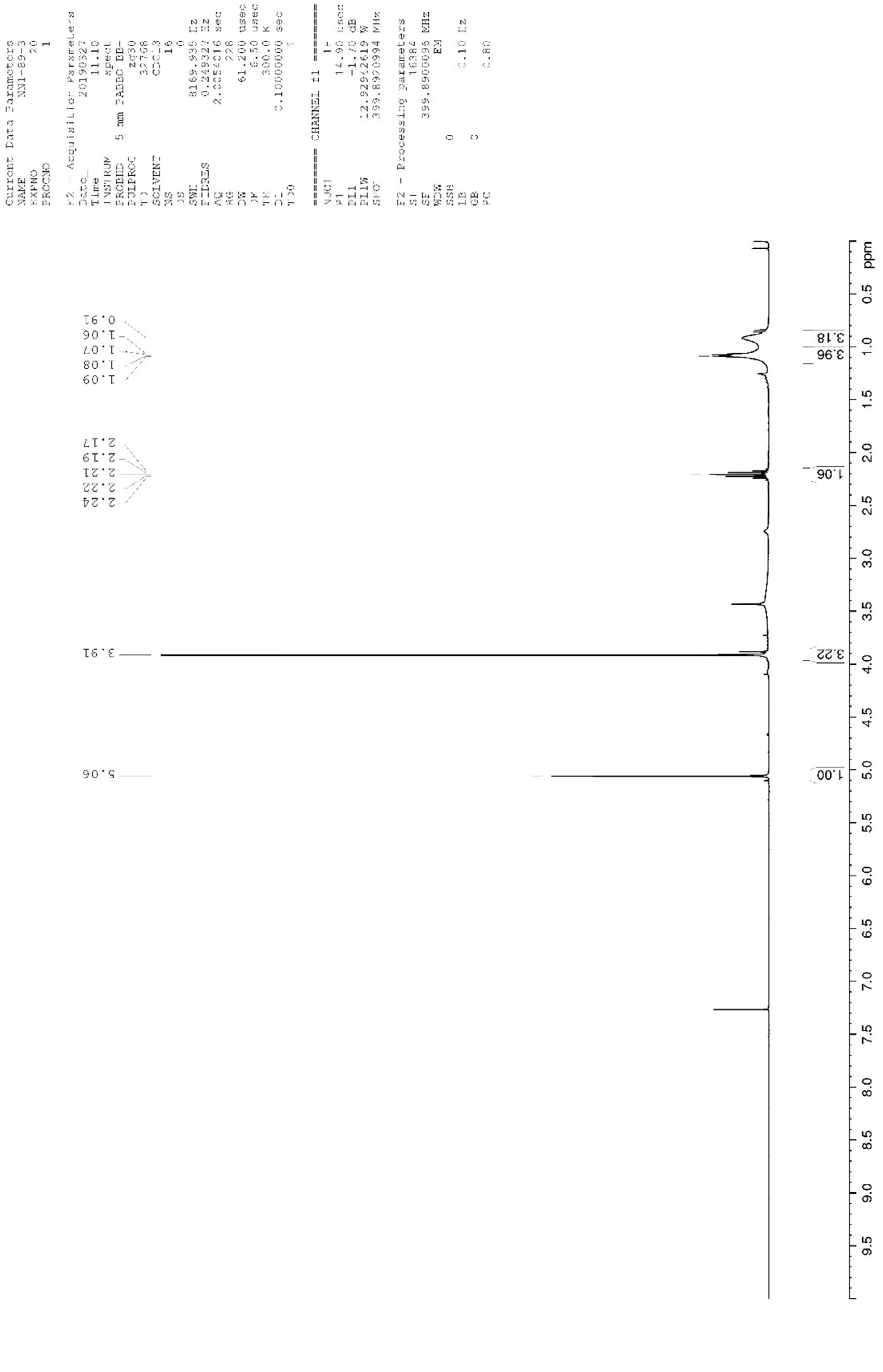

Figure S8. ^{1}H NMR spectrum (400 MHz, $CDCl_3$) of **1b**

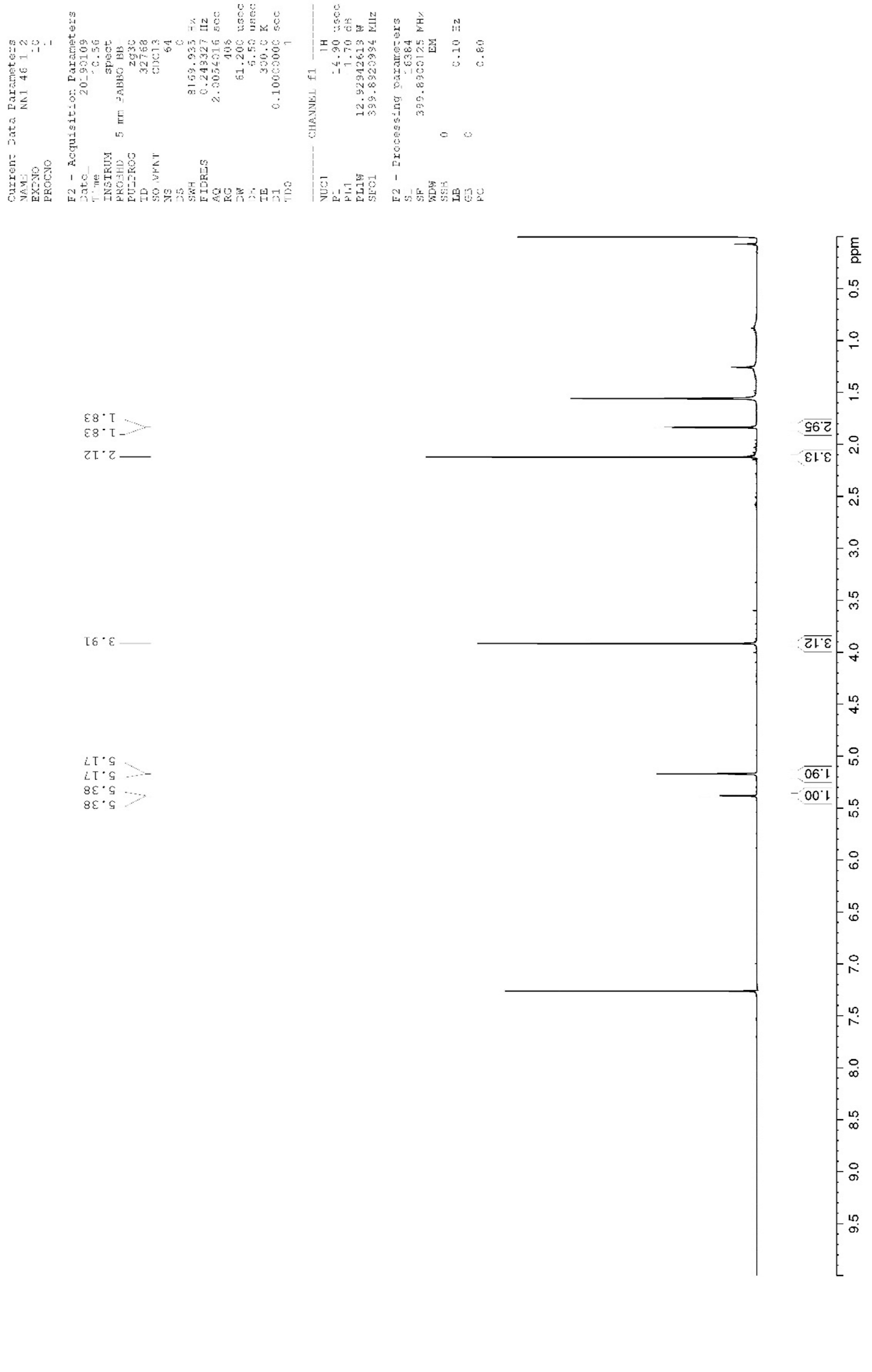

Figure S9. ^{1}H NMR spectrum (400 MHz, $CDCl_3$) of **1c**

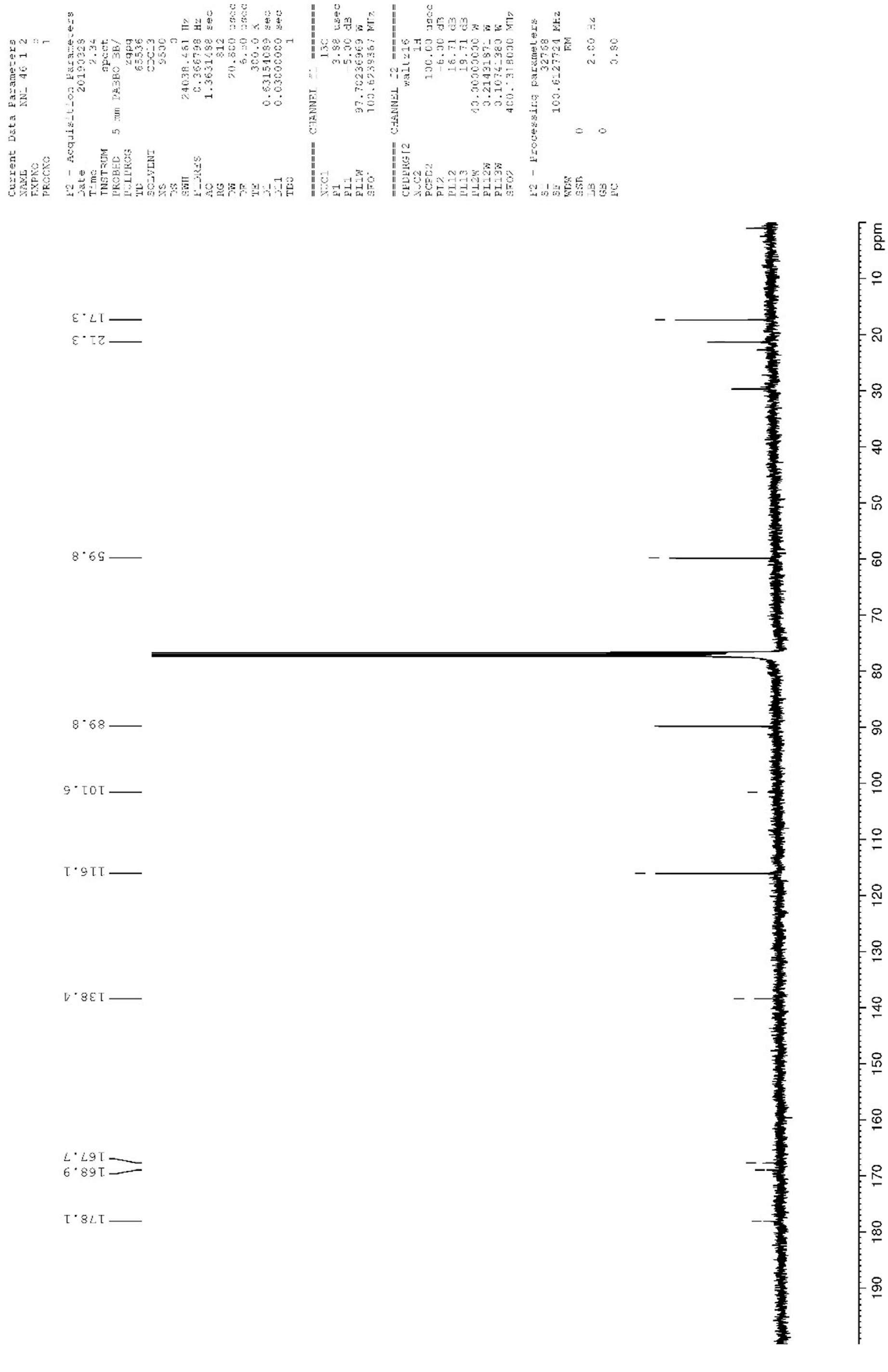

Figure S10. ^{13}C NMR spectrum (100 MHz, $CDCl_3$) of **1c**
